# Second-stage non-response in the Swiss health survey: determinants and bias in outcomes

**DOI:** 10.1186/1471-2458-13-167

**Published:** 2013-02-23

**Authors:** Thomas Volken

**Affiliations:** 1 Zurich University of Applied Sciences, School of Health Professions, Technikumstr. 71, P.O. Box, CH-8401, Winterthur, Switzerland

## Abstract

**Background:**

Unit non-response occurs in sample surveys when a target subject does not respond to a survey. Potential implications are decreased power, increased standard error, and non-response bias. The objective of this study was to assess the factors associated with participation in a written survey (MSHS) of subjects who had previously participated in the Swiss Health Survey (SHS) and to evaluate to what extent non-participation could impact the estimation of various MSHS health outcomes.

**Methods:**

Multivariate logistic regression was used to assess the factors associated with MSHS participation (n=14,393) by eligible SHS participants (n=17,931). Crude participation rates and the adjusted odds ratios of participation (OR) were reported. In order to report potential bias in MSHS outcomes, the average age-standardized and sex-specific outcome values in non-participants were predicted based on several different linear regression models which had been previously fitted on MSHS participants.

**Results:**

Adjusting for all other variables, women (OR=1.63) as compared with men, subjects with a secondary (OR=1.48) or tertiary education (OR=1.76) as compared with those with primary education, white-collar workers (high level non-manual workers OR=1.29, medium and low level non-manual workers OR=1.26 and OR=1.25 respectively) as compared with unskilled manual workers, Swiss nationals (OR=1.60) as compared to non-Swiss, and subjects with very good or good self-rated health (OR=1.35) were more likely to participate in the MSHS. People who work full-time were less likely to participate than those without paid work (OR=0.76). There were no statistically significant differences in the likelihood of participation between rural and urban areas, different geographic regions of Switzerland and household income quartiles.

Except for myocardial infarction, all age-standardized and sex-specific average outcomes (influenza vaccination, arthrosis, osteoporosis, high blood pressure, depression, mastery, and sense of coherence) were significantly different between MSHS non-participants and participants.

**Conclusions:**

Subjects who participated in the MSHS had a higher socio-economic status, reported a better subjective health, and were more likely to be Swiss nationals. Small to moderate bias was found for most age-adjusted and sex-specific average outcomes. Consequently, these MSHS outcomes should be used and interpreted with care.

## Background

Unit non-response occurs in sample surveys when a target subject does not respond to a survey. Potential implications of unit non-response are decreased power due to reduced sample size, increased standard error due to random error, and non-response bias due to systematic error because the calculation of estimates is generally based on those who responded to the survey. Consequently, the handling of unit non-response [1-7], the determinants of non-response [8-13] and its effect on results of interest [9,14-18] have become a major topic of interest [2]. More specifically, several studies have been undertaken on the non-response to health interview surveys (HIS). Previous studies find that respondents have a higher socio-economic status and that they report a better subjective health, lower healthcare use, and healthier lifestyle behaviour than non-respondents [19-28]. However, other studies find higher healthcare use for respondents or better health status for non-respondents [29-33]. So far, few studies have investigated selection mechanisms in second-stage non-response in HIS designs [22,34]. At the second stage, HIS participants are asked to participate in yet another survey. At this stage, the population has already been willing to give personal information on their health, their health service utilization or their health-related behavior. Hence, their characteristics, their motives and the underlying mechanisms of non-response may be different from those of subjects who refuse to participate in the initial interview. While little is known about second-stage non-response in general, there is currently no study that investigates second-stage non-response in the Swiss Health Survey (SHS). The SHS is the main cross-sectional, population-based HIS in Switzerland which intends to monitor health trends in a representative sample of permanent residents. At the end of a lengthy face to face or telephone interview, eligible SHS participants (n=17,931) were asked to complete and return an additional mail survey (MSHS). The mail-back questionnaire was returned by 80.3% of the interviewees. Since extensive data were available on all SHS participants, no matter whether they participated in the MSHS or not, the current study is able to assess second-stage non-response in the SHS. The objectives of this study are: 1) to describe the factors associated with MSHS participation and 2) to assess to what extent non-participation could impact the estimation of major health outcomes monitored by the MSHS.

## Methods

### Study design

The study is a methodologically-focused secondary analysis of a population-based, cross-sectional health survey carried out in 2007 in Switzerland.

### Study population and data

The Swiss Health Survey carried out by the Swiss Federal Statistical Office (SFSO) is a nationwide survey on health status, health service utilization, and health-related behavior in Switzerland. The SHS was first conducted in 1992 and is repeated every five years. In 2007, a multistage probability sample was drawn of the entire Swiss population after stratification by geographic region. The initial sample (n=28,319) included subjects aged 15 years or older living in private households. The sampling frame excluded people living in institutions, i.e. hospitals, homes for the elderly, prisons, monasteries, barracks, etc. Furthermore, only subjects fluent in German, French or Italian were included. The response rate of those in the initial sample was 66.2% (n=18’760); 13.2% (n=3^′^730) could not be contacted after numerous contact attempts, and 20.6% (n=5’825) refused to participate in the SHS. Of the latter, 445 subjects refused to participate due to language problems (1.6% of the initial sample).

Three different types of interviews were conducted. Generally, computer-aided telephone interviews (CATI) were conducted for subjects aged 15–74 years (n=17,856). Subjects aged 75 years or older were given the choice between a CATI or a face to face interview. For those opting for face to face interviews, computer-aided personal interviews (CAPI) were conducted (n=75). Finally, CATI were conducted with proxy persons (PROXY, n=829), that is close friends or relatives of the target subject were interviewed if the target subject could not be interviewed because of old-age, illness, handicap, language problems or due to absence from home for more than 4 weeks. At the end of the forty-minute interview, CATI and CAPI participants (n=17,931) were invited to complete and return an additional written survey (MSHS). PROXY participants were not eligible to participate in the MSHS. Of those eligible to participate, 97.7% (n=17’511) consented to return the MSHS and the questionnaires were sent out to those willing to participate in the next 2–3 days following the SHS interview. The mail-back questionnaire which included questions on mental stress, mental illness, chronic diseases, vaccination status, social support, health literacy, health insurance coverage etc., was returned by 80.3% (n=14,393) of the 17,931 CATI & CAPI interviewees. Subjects who did not return the questionnaire were not re-contacted.

### Imputation

In order to prevent the loss of information and to prevent potential bias due to selective refusal to provide answers, item non-response was addressed by the statistical technique of multiple imputations (MI) and multivariate normal regression was used as the imputation method to estimate missing values [35-38]. In contrast to other imputation procedures, MI is more likely to yield valid statistical inference than other methods of imputation because its simulation-based procedures treat imputed values as estimates with a random component rather than treating the imputed values as known with certainty. For SHS variables, the percentage of imputed values ranged from 0.04% to 5.64%. For MSHS variables, between 1.74% and 9.73% of the values were imputed (Table 1). To obtain estimates based on the pooled set of imputation results from each completed dataset (m=20), subsequent statistical analyses were carried out using Stata’s “mi estimate” prefix.

**Table 1:** Frequency of imputed values in SHS and MSHS

	**Source**	**Complete**	**Imputed**	**Total**	**%-imputed**
Occupational status	SHS	17,083	848	17,931	4.73
Employment	SHS	17,543	388	17,931	2.16
Income	SHS	16,920	1,011	17,931	5.64
Nationality	SHS	17,921	10	17,931	0.06
Religious denomination	SHS	17,846	85	17,931	0.47
Self-rated health	SHS	17,923	8	17,931	0.04
Body Mass Index	SHS	17,704	227	17,931	1.27
Mental Health Inventory	SHS	17,634	297	17,931	1.66
Depression CIDI-SF	SHS	17,855	76	17,931	0.42
Mastery	MSHS	12,992	1,401	14,393	9.73
Sense of coherence	MSHS	13,548	845	14,393	5.87
Influenza vaccination	MSHS	14,143	250	14,393	1.74
Arthrosis	MSHS	13,395	998	14,393	6.93
Osteoporosis	MSHS	13,328	1,065	14,393	7.40
High blood pressure	MSHS	13,568	825	14,393	5.73
Myocardial infarction	MSHS	13,341	1,052	14,393	7.31
Depression	MSHS	13,388	1,005	14,393	6.98

Data source: Swiss Federal Statistical Office.

### Statistical analyses

Multivariate logistic regression was used to assess the factors associated with MSHS participation. Crude participation rates and the adjusted odds ratios of participation were reported. The latter were adjusted for age (including age^2^), gender, the interaction between age and gender, socio-economic status (including educational level, occupational status, employment, and income), nationality, religious denomination, urbanity, geographic region, self-reported health and the number of contact attempts needed until the CATI or CAPI could be realized. Age was measured in years (whole-numbers), contact attempts were also represented in continuous whole-numbers. The remaining covariates comprised two or more categories: gender (female, male), educational level (primary, secondary, and tertiary education), occupational status (high level non-manual, medium level non-manual, low level non-manual, self-employed craftsmen, skilled manual, unskilled manual), employment (full time, part time, not in workforce), income (1^st^, 2^nd^, 3^rd^, and 4^th^ quartile of household income), nationality (Swiss, non-Swiss), religious denomination (Protestant, Free Protestant, Roman Catholic, Christian Catholic, Christian Orthodox, other Christian, Jewish, Muslim, other, none). Urbanity comprised two categories: rural area and urban area. Urban areas included isolated cities (≥10,000 inhabitants) and urban agglomerations (≥20,000 inhabitants); all other areas were assigned to the category rural area. Geographic region comprised the seven greater regions of Switzerland (Midlands, Northwest, Zurich, East, Central, Tessin, and Lake Geneva Region). Self-reported health comprised two categories (very good or good health versus moderate, bad or very bad health).

To assess potential bias in MSHS outcomes, 8 variables from 3 domains (chronic diseases, prevention, psycho-social resources) were selected and the average outcome values in non-participants were predicted based on several different linear regression models which had been previously fitted on MSHS participants. From the 14 chronic diseases covered by the MSHS, 5 chronic diseases were selected based on the frequency of item non-response, i.e. outcomes with fewer missing values were preferred. The 5 disease related outcomes comprised the lifetime prevalence of arthrosis, osteoporosis, high blood pressure, myocardial infarction, and depression. Only 2 outcomes related to prevention were covered by the MSHS: contraception and influenza vaccination. The 12 month prevalence of influenza vaccination was selected because of its relevance in the contemporary Swiss public health debate, i.e. Switzerland faces a new law on mandatory vaccination. Finally, mastery and sense of coherence, the two psycho-social resources covered by the MSHS were selected because of their relevance when coping with stressful life events such as diseases [39]. While the mastery instrument was based on a brief version of the Pearlin coping questionnaire [40], the SOC instrument was based on a brief version of Antonovsky’s sense of coherence questionnaire [41]. All eight prediction models included socio-economic and sociodemographic covariates (age, gender, educational level, occupational status, employment, income, nationality, religious denomination, urbanity and geographic region) as well as self-reported health (all indicators measured as described above) and the Mental Health Inventory score (MHI-5). The MHI-5 is a 5-item subscale from the SF-36 [42,43] which is used to capture four major dimensions of mental health: anxiety, depression, loss of behavioural/emotional control, and psychological well-being.

Outcome estimates related to the lifetime prevalence of arthrosis, osteoporosis, high blood pressure, myocardial infarction, and depression also included the body mass index (BMI) as a covariate. BMI calculation was based on self-reported height and weight (BMI=kg/m^2^). Finally, the existence of an acute major depression according to DSM-IV criteria (measured using CIDI-SF [44]) was added as a binary covariate to the model predicting the lifetime prevalence of depression. Crude average outcomes and age-standardized, sex-specific average outcomes were compared between participants and non-participants of the MSHS. Statistical significance was established at p ≤ 0.05.

## Results

### Factors associated with participation

**Figure 1:**
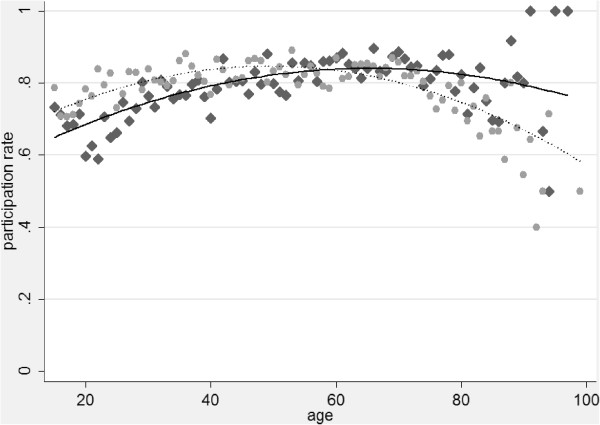
**MSHS participation rates among eligible CATI&CAPI participants by gender and age.** Diamonds, crude rates for men; circles, crude rates for women; solid line, fitted values for men; dotted line, fitted values for women. Fitted values are adjusted for all variables in Table 2, the interaction term gender x age, and the number of contact attempts needed to realize the CATI/CAPI. Data source: Swiss Federal Statistical Office.

Overall, more than 80% of the subjects who completed the SHS CATI or SHS CAPI also completed and returned the MSHS (Table 2). Participation rates were relatively high in all groups and never dropped below 60%. However, substantial differences between socio-economic as well as between sociodemographic groups were found. Generally, people from lower socio-economic strata exhibited lower participation rates. Similarly, non-Swiss nationals and members of some religious denominations showed lower participation rates. Participation rates varied also with age and their patterns were different between women and men (Figure 1). When adjusting for all other variables, these initial results were confirmed. Participation rates of both, men and women, increased gradually with age, eventually reached their maximum and decreased with age thereafter. The participation rates of women started to decrease at approximately 45 years of age, the participation rates of men began to decrease at approximately 60 years of age. At younger ages, the participation rates of women were generally higher than those of men whereas participation rates of men were equal to those of women at approximately 56 years of age and started to be higher than those of women at older ages. Overall, women were more likely to participate in the MSHS than men (OR=1.63), age^2^ (OR=0.99), age (OR=1.06), and the interaction between age and gender (OR=0.99) were all significantly associated (p<0.001) with MSHS participation. People with a secondary (OR=1.48) or tertiary education (OR=1.76) as compared with people with primary education were more likely to participate in the MSHS as were white-collar workers (high level non-manual workers OR=1.29, medium and low level non-manual workers OR=1.26 and OR=1.25 respectively) as compared with unskilled manual workers. People who work full-time were less likely to participate than those without paid work (OR=0.76), and Swiss nationals were more likely to participate than were non-Swiss nationals (OR=1.60). Compared with people who had no religious affiliation, members of the Christian Catholic Church (OR=0.66), the Orthodox Church (OR=0.49), Muslim religious communities (OR=0.36) or the residual category *other religious community* (OR=0.52) were less likely to participate in the MSHS. Members of the remaining religious denominations, most notably members of the large Swiss State Churches (Protestant and Roman Catholic Church), did not differ from people without religious affiliation. People with better self-rated health (OR=1.35) were also more likely to complete and return the MSHS as compared with people who rated their health as moderate, bad or very bad.

**Table 2:** **MSHS participation rates among eligible CATI** &CAPI participants and multivariate odds ratios of participating in the MSHS^*^

**Variable**	**Category**	**n**	**Rate (%)**	**OR**	**95%-CI**	**P-value**
All	All MSHS participants	14,393	80.27			
Gender	Female	8,085	80.96	1.63	1.31, 2.03	<0.001
	Male	6,308	79.40	1.00	reference	
Educational level	Tertiary education	3,970	84.63	1.76	1.52, 2.04	<0.001
	Secondary education	8,689	80.63	1.48	1.32, 1.65	<0.001
	Primary education	1,734	70.37	1.00	reference	
Occupational status	High level non-manual	1,390	84.67	1.29	1.06, 1.55	<0.010
	Medium level non-manual	4,431	83.46	1.26	1.10, 1.43	<0.010
	Low level non-manual	3,738	81.78	1.25	1.10, 1.42	<0.010
	Self-employed craftsmen	1,168	77.46	0.85	0.72, 1.00	0.052
	Skilled manual	1,500	73.62	0.92	0.80, 1.06	0.243
	Unskilled manual	2,162	75.51	1.00	reference	
Employment	Full time	5,360	77.93	0.76	0.68, 0.85	<0.001
	Part time	3,599	84.69	1.03	0.91, 1.17	0.635
	Not in workforce	5,434	79.88	1.00	reference	
Quartile of household income	4^th^ (highest)	3,690	82.36	1.04	0.92, 1.18	0.547
	3^rd^	3,622	82.09	1.08	0.96, 1.22	0.185
	2^nd^	3,631	79.87	1.02	0.91, 1.14	0.789
	1^st^ (lowest)	3,451	76.80	1.00	reference	
Nationality	Swiss	12,925	81.95	1.60	1.43, 1.79	<0.001
	Non-Swiss	1,468	68.01	1.00	reference	
Religious denomination	Protestant	5,064	83.59	1.07	0.94, 1.22	0.300
	Free protestant	149	80.54	0.95	0.65, 1.41	0.816
	Roman catholic	6,379	79.50	0.88	0.79, 1.01	0.063
	Christian catholic	263	71.47	0.66	0.51, 0.86	<0.010
	Orthodox	118	61.46	0.49	0.36, 0.68	<0.001
	Other Christian	69	77.53	0.76	0.45, 1.27	0.292
	Jewish	39	79.59	0.87	0.42, 1.79	0.703
	Muslim	145	50.17	0.36	0.27, 0.46	<0.001
	Other	142	68.27	0.52	0.38, 0.72	<0.001
	None	2,027	81.99	1.00	reference	
Urbanity^1)^	Rural area	4,575	80.65	1.02	0.93, 1.12	0.649
	Urban area	9,818	80.09	1.00	reference	
Geographic region	Midlands	3,822	81.32	1.08	0.96, 1.21	0.226
	Northwest	1,546	80.73	1.00	0.87, 1.16	0.950
	Zurich	1,867	79.85	0.92	0.80, 1.06	0.241
	East	1,558	81.15	1.09	0.94, 1.26	0.259
	Central	1,856	80.03	1.03	0.90, 1.18	0.680
	Tessin	1,131	78.54	1.08	0.92, 1.27	0.345
	Lake Geneva Region	2,613	79.21	1.00	reference	
Self-rated health	Very good, good	12,491	81.14	1.35	1.21, 1.50	<0.001
	Very bad, bad, moderate	1,902	74.97	1.00	reference	

^*^n: number of subjects in category;%: participation rate within category; OR: odds ratios, adjusted for all other variables in the table as well as age, interaction term gender x age, and the number of contact attempts needed to realize the CATI/CAPI; 95%-CI: 95% confidence intervals; P-Value: P>|t|.

^1)^ Urban areas include isolated cities (≥10,000 inhabitants) and urban agglomerations (≥20,000 inhabitants). Data source: Swiss Federal Statistical Office.

Finally, no statistically significant differences in the likelihood of participation were observed between rural and urban areas, different geographic regions of Switzerland and household income quartiles.

### Potential bias in outcomes

In order to assess potential bias in MSHS outcome variables, outcomes of non-participants were estimated based on different linear regression models which had been previously fitted on MSHS participants. Table 3 shows the model fit as well as the strongest predictors (P<0.001) for each outcome variable. Generally, the percentage of variance explained by the models was moderate and ranged from 18.6% to 26.7%.

**Table 3:** **Predictors with P**<**0**.**001 in the models for MSHS outcomes**

**Outcome**	**Predictors with P <0**.**001**	**Variance predicted (%)**
Influenza vaccination	Age, occupational status, MHI-5, self-rated health, geographic region	21.2
Arthrosis	Age, sex, MHI-5, self-rated health, BMI	21.7
Osteoporosis	Age, sex, MHI-5, self-rated health, BMI	25.0
High blood pressure	Age, MHI-5, self-rated health, BMI	26.7
Myocardial infarction	Age, sex, self-rated health	21.2
Depression	Age, employment, MHI-5, self-rated health, depression CIDI-SF	18.6
Mastery	Age, occupational status, MHI-5, self-rated health, geographic region	24.8
Sense of coherence	Age, sex, occupational status, MHI-5, self-rated health, religious denomination	26.5

Data source: Swiss Federal Statistical Office.

Crude predicted values for the MSHS non-participants (Table 4) were higher than the upper boundary of the 95% confidence interval (95%-CI) of the MSHS participants in four of the eight outcomes (influenza vaccination, arthrosis, high blood pressure, and depression) and they were lower than the lower boundary of 95%-CI of the MSHS participants in two outcomes (mastery and SOC). The predicted lifetime prevalence of osteoporosis and myocardial infarction of non-participants was within the boundaries of the 95%-CI of MSHS participants. Age-standardized and sex-specific figures (Table 5) give a more differentiated picture. For male non-participants, the predicted 12 month prevalence of influenza vaccination, the lifetime prevalence of arthrosis, osteoporosis, and high blood pressure were found to be lower than the lower boundary of the 95%-CI of male MSHS participants. For female non-participants, the corresponding predicted outcomes were higher than the upper boundary of the 95%-CI of female MSHS participants. The predicted lifetime prevalence of depression of female and male non-participants was higher than the upper boundary of the corresponding 95%-CI of MSHS participants. Finally, average mastery and SOC scores of male and female non-participants were lower than the lower boundary of the 95%-CI of corresponding male and female participants.

**Table 4:** **Outcomes in MSHS participants and predicted outcomes in non**-participants^*^

**Outcome**	**Mean value or %**	**95%-CI**	**Predicted value**
Influenza vaccination (%)	19.05	18.41, 19.70	19.81
Arthrosis (%)	15.45	14.83, 16.07	16.28
Osteoporosis (%)	3.73	3.41, 4.06	3.90
High blood pressure (%)	18.92	18.26, 19.58	19.69
Myocardial infarction (%)	2.31	2.06, 2.56	2.50
Depression (%)	9.18	8.99, 9.37	10.30
Mastery (mean)	13.27	13.22, 13.31	13.02
Sense of coherence (mean)	17.09	17.04, 17.14	16.65

^*^95%-CI: 95% confidence intervals. Data source: Swiss Federal Statistical Office.

**Table 5:** **Age**-**standardized and gender**-**specific outcomes in MSHS participants and predicted outcomes in non**-participants^*^

	**Men**			**Women**		
**Outcome**	**Mean value or %**	**95%-CI**	**Predicted value**	**Mean value or %**	**95%-CI**	**Predicted value**
Influenza vaccination (%)	16.52	15.60, 17.44	14.82	16.41	15.58, 17.23	17.56
Arthrosis (%)	10.63	9.85, 11.42	9.57	16.48	15.63, 17.34	18.45
Osteoporosis (%)	0.74	0.52, 0.96	0.12	5.17	4.68, 5.66	5.97
High blood pressure (%)	17.86	16.89, 18.83	16.20	16.04	15.21, 16.88	17.92
Myocardial infarction (%)	2.94	2.52, 3.36	2.94	1.05	0.83, 1.28	1.08
Depression (%)	7.23	6.57, 7.89	8.64	11.17	10.45, 11.88	12.54
Mastery (mean)	13.36	13.30, 13.43	13.08	13.15	13.09, 13.21	12.88
Sense of coherence (mean)	17.06	16.98, 17.13	16.53	17.08	17.01, 17.15	16.70

^*^95%-CI: 95% confidence intervals. Source: Swiss Federal Statistical Office.

## Discussion

More than 80% of eligible SHS participants were willing to complete and return the additional mail-back questionnaire.

Subjects who participated in the mail survey had a higher socio-economic status (higher education, white-collar workers), reported a better subjective health and were more likely to be Swiss nationals. Generally, these finding are in accordance with previous studies that investigated initial non-response [3,19-28]. Unfortunately, a rigorous comparison of initial and second-stage SHS/MSHS non-response is not feasible because systematic data on non-response in the SHS are unavailable. However, it is plausible that the mechanisms of initial non-response in the SHS are not very different from those found in previous HIS studies, at least when it comes to important sociodemographic and socioeconomic factors such as gender, education, employment and nationality. Moreover, available population data in comparison with SHS/MSHS data (Table 6) show that the proportion of men, the proportion of subjects with primary education, the proportion of subjects with full-time employment, and the proportion of non-Swiss is lower in the SHS and MSHS samples. At least partially, these differences may be attributed to non-response. Together, evidence from previous HIS non-response studies and available population data suggest that there seems to be no fundamental difference between the mechanisms of initial non-response and second-stage non-response. Consequently, the systematic under-representation of the same group or groups of non-respondents is reinforced in the second stage. In the MSHS, this may be specifically the case for non-Swiss nationals with low educational levels. While crude MSHS response rates for Swiss nationals were 73.4% for subjects with primary education, 82.2% for subjects with secondary education, and 86.7% for subjects with tertiary education, the respective response rates for non-Swiss nationals were substantially lower (58.3%, 66.4%, and 78.3%). Furthermore, the differences between Swiss and non-Swiss nationals were much more pronounced in the lower educational levels. Unfortunately, no information is available on the initial SHS response rates by nationality and educational level. However, it is very likely that non-Swiss nationals had lower SHS response rates since interviews were conducted in German, French or Italian which potentially favoured non-Swiss nationals who are well-integrated, well-educated and have been living in Switzerland for a long period of time. Given that in 2009, non-Swiss nationals accounted for 1.7 million people or roughly 22% of the permanent resident population of Switzerland, the implications of lower response rates in this group should not be taken lightly.

**Table 6:** **Proportions of selected demographic variables in the Swiss permanent resident population and in the SHS**/**MSHS**

**Variable**	**Category**	Population^*)^	SHS sample^*)^	MSHS sample^*)^
Gender	Female	50.7	55.7	56.2
	Male	49.3	44.3	43.8
Educational level^1)^	Tertiary education	31.3	32.3	33.4
	Secondary education	54.7	58.4	58.7
	Primary education	14.0	9.4	7.9
Employment^2)^	Full Time	54.9	49.4	48.0
	Part Time	27.9	28.5	30.1
	Not in workforce	17.2	22.1	21.9
Nationality	Non-Swiss	22.0	12.0	10.2
	Swiss	78.0	88.0	89.8

^*)^ percent values; ^1)^ subjects aged 24–64 years; ^2)^ subjects aged 15–64 years.

Data source: Swiss Federal Statistical Office.

Subjects without paid work and subjects who worked part-time were more likely to participate in the MSHS than those who worked full-time (all other things being equal). Although this finding is not in accordance with previous evidence [22], one plausible explanation is that subjects who work full-time have lower time budgets and are therefore less willing to spend time on completing and returning the questionnaire.

Religious affiliation was also found to be associated with MSHS non-response. Especially subjects who were members of relatively small religious denominations were less likely to return the mail-back questionnaire while the response rates of members of the large Swiss State Churches (Protestant Church, Roman Catholic Church) and subjects with no religious affiliation did not significantly differ from each other. These finding are in partial agreement with one previous study of second-stage non-response which observed that Muslims had lower response rates than members of other religious denominations [19].

MSHS response rates were not different between rural and urban areas and between different geographic regions. Previous evidence is mixed: some studies observed that response rates are higher in rural areas [13,45,46], others found that response rates are higher in urban areas [22,47]. In Switzerland, the presence of similar response rates in rural and urban areas may be related to geographic proximity. Switzerland has a very dense network of post offices. Therefore, travel time to the nearest post office to mail back the questionnaire is hardly an issue, if at all.

Finally, numerous previous studies report that females are more likely to participate in surveys [16,21,24,28,45,48]. While the current study also found higher female participation, the current study did not find a uniform pattern of higher female participation in the MSHS. Rather, participation rates of men and women were linked with age, i.e. at younger ages, the participation rates of women were higher than those of men whereas participation rates of men were higher at older ages. The fact that higher male participation rates approximately coincide with retirement age may suggest that men are more willing than women to invest their additional time resources in the completion of surveys. This hypothesis should be examined in further studies.

In a second step, the current study attempted to quantify the magnitude of the bias due to selective non-response by estimating population average values of eight major MSHS outcome variables. Although overall non-participation rates were relatively low, non-participation was clearly associated with socio-economic status, non-Swiss nationality, and health status. Hence bias is potentially induced by the under-representation of non-Swiss nationals and lower socio-economic strata. Bias could be demonstrated for all crude average outcomes, except for the lifetime prevalence of osteoporosis and myocardial infarction. Furthermore, bias could also be shown for all age-standardized and sex-specific average outcomes, except for myocardial infarction. The predicted age-standardized average mastery and SOC scores of male and female MSHS non-respondents were below the respective values for respondents while the predicted lifetime prevalence of depression was higher in non-respondents. These results support the notion that bias is potentially induced by the under-representation of lower socio-economic strata: earlier studies find that lower mastery and SOC scores are associated with lower socio-economic status [49,50] and higher SOC and mastery scores are associated with a better mental health status [50-52]. There is also evidence that some immigrant groups in Switzerland have higher hospitalization rates due to affective disorders as compared to Swiss nationals [53]. Similarly, the Swiss Migrant Health Survey 2010 reports better health for Swiss nationals as compared to nationals from Portugal, Serbia, Kosovo, and Turkey. More specifically, Swiss nationals were less likely to suffer from depression, high blood pressure, arthrosis, osteoporosis, sick headache, and allergy than most non-Swiss nationals [54].

Bias was also observed for age-standardized and sex-specific average lifetime prevalence of high blood pressure, arthrosis, and osteoporosis. However, the predicted prevalence rates for female non-participants were above those for female MSHS participants while the predicted prevalence rates for male non-participants were below those for male participants. This may be due to a gender-specific self-selection mechanism: subjects with high socio-economic status (especially tertiary education and high and medium level non-manual workers) were much more common in male than in female non-participants.

Although bias could be demonstrated for several MSHS outcomes, the magnitude of the bias is at most moderate, i.e. the differences between age-standardized and sex-specific average prevalence rates of MSHS respondents and predicted prevalence rates of non-respondents are below 2% and the respective differences in average mastery and SOC scores cannot be considered substantial. Yet, the SHS response rate amounted to little more than 66% which suggests that SHS participants may be a selective sample of the general population of Switzerland. Moreover, the SHS sampling frame excluded subjects living in institutions and included only subjects fluent in German, French or Italian. Were these subjects part of the SHS, their non-participation may further increase bias because they may have high MSHS non-response rates and poorer health.

The current study has important limitations. Firstly, prevalence calculations for non-respondents are based on parameter estimates for respondents. This implies that the selected prevalence models for respondents also yield valid results for non-respondents. However, given the various documented differences between respondents and non-respondents, this may not necessarily be the case. That is, the use of models suited for respondents may be a potential source of bias when used to estimate the prevalence rates for non-respondents. Secondly, systematic data on initial non-response in the SHS were unavailable. Hence, the discussion on initial and MSHS second-stage non-response had to be based on previous evidence on initial HIS non-response and the available population data.

## Conclusions

Subjects who participated in the MSHS had a higher socio-economic status (higher education, white-collar workers), reported a better subjective health and were more likely to be Swiss nationals. Small to moderate bias was found for most age-adjusted and sex-specific average outcomes, i.e. the prevalence of depression, arthrosis, osteoporosis, high blood pressure, and influenza vaccination as well as the Pearlin mastery and sense of coherence scores. Consequently, these MSHS outcomes should be used and interpreted with care. Further waves of the SHS/MSHS should especially improve participation of non-Swiss and subjects with low socio-economic status. Furthermore, the SHS should include variables that facilitate a more rigorous investigation of initial non-response and thus facilitate a better understanding of non-response bias.

## Competing interests

The author declares that he has no competing interests.

## Authors’ contributions

TV contributed all of the writing and the analysis.

## Pre-publication history

The pre-publication history for this paper can be accessed here:

http://www.biomedcentral.com/1471-2458/13/167/prepub
